# Hematoma Disguised as Cancer

**DOI:** 10.7759/cureus.39665

**Published:** 2023-05-29

**Authors:** Alec Garfinkel, Alex Nagourney, Michael C Larson

**Affiliations:** 1 Radiology, California Northstate University College of Medicine, Elk Grove, USA; 2 Internal Medicine, California Northstate University College of Medicine, Elk Grove, USA; 3 Radiology, University of California – Davis, Sacramento, USA

**Keywords:** contrast-enhanced ultrasound, mri abdomen, body mri, digital subtraction angiography(dsa), ct abdomen and pelvis with iv contrast, liver, infra-hepatic hematoma

## Abstract

Hematomas are often associated with benign processes such as sport-related injuries, postsurgical complications, and medications such as blood thinners. Rarely, hematomas can occur spontaneously without any identifiable cause or recollection of an inciting event. Such events can lead to inaccurately diagnosing a patient, which could delay or alter treatment and worsen the patient’s prognosis. This patient reported sudden-onset abdominal pain with radiation to her back and denied any recent medication use or trauma while at home. The case highlights the key radiographic findings of magnetic resonance imaging (MRI) and contrast-enhanced ultrasound to eventually confirm an obscure case of hepatocellular carcinoma and help guide management.

## Introduction

Spontaneous subhepatic hematoma is an uncommon yet life-threatening condition with only a few cases reported in the literature [1]. Many cases are the result of complications from preeclampsia or hemolysis, elevated liver enzymes, and low platelet count (HELLP) syndrome during pregnancy. Other differential diagnoses including metastatic disease and rupture of focal nodular hyperplasia, hepatic adenoma, hepatic artery aneurysm, and hepatocellular carcinoma (HCC) are less common but should also be considered. Due to the high morbidity and mortality of the condition, a thorough work-up is warranted following the stabilization of the patient. Atraumatic hepatic hematoma is often diagnosed with a combination of imaging modalities including abdominal ultrasonography (US), magnetic resonance imaging (MRI), and computerized tomography (CT) [2]. This case demonstrates the utility of contrast-enhanced ultrasound (CEUS) Liver Imaging Reporting and Data System (LI-RADS) along with CT/MRI LI-RADS by presenting a case with radiographic findings consistent with malignancy in the setting of HCC rupture.

## Case presentation

A 69-year-old woman with hepatitis B presented to the emergency department with acute-onset abdominal pain. The patient was at home and sitting on her couch when she first noticed the pain. The pain was sudden in onset, constant aching, and localized to her right upper quadrant with 9/10 severity but radiating to the right side of her back. Movement, eating, and lying down did not exacerbate the pain. She did not attempt taking over-the-counter pain medications for her abdominal pain in the short course between onset and presenting to the emergency department. She endorsed dyspnea with generalized weakness but denied other issues on review of symptoms and history, including no recent trauma or sick contacts. The patient had never experienced this type of abrupt-onset abdominal pain in the past. Other than hepatitis B presumed dormant for the past 20 years, the patient had no significant medical history. Her family history was unremarkable. Although she drank 1-2 alcoholic beverages on the weekends, she denied tobacco and recreational drug use and reported eating a well-balanced diet. On physical examination, the patient was alert and oriented though ill-appearing with acute distress, pale, and diaphoretic. Lungs were clear to auscultation, and she was tachycardic with sinus rhythm. There was abdominal tenderness to palpation along with rebound tenderness diffusely. Bowel sounds were heard, and her abdomen was tympanic in all four quadrants. No bruits nor palpable abdominal aortic aneurysms were detected.

**Figure 1:**
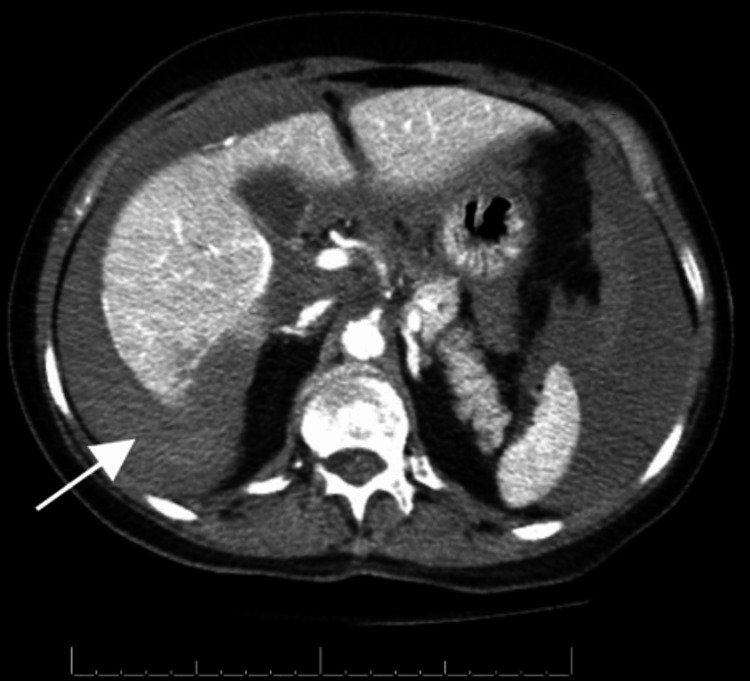
Representative axial portal venous phase CT image on admission showing hemoperitoneum (white arrow). CT: computed tomography.

**Figure 2:**
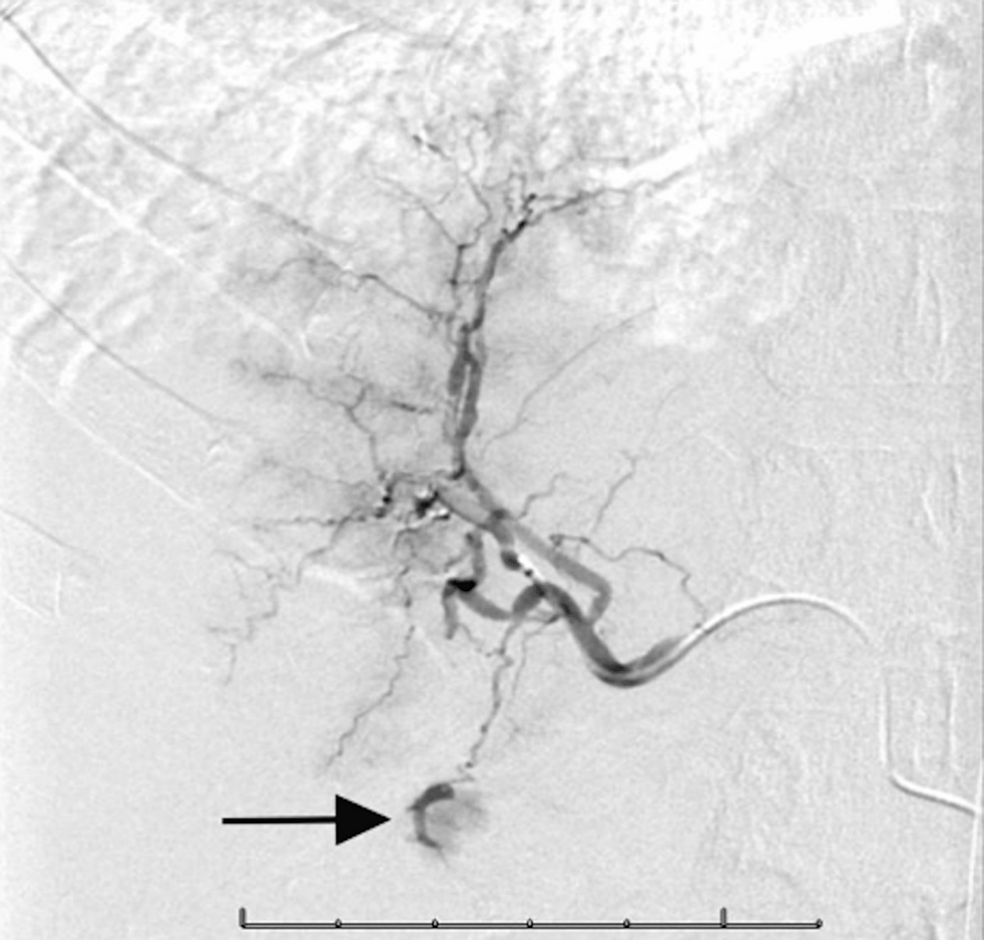
Admission DSA showing selective common hepatic artery angiogram with abnormal pooling of the right hepatic artery lobe branch (black arrow). DSA: digital subtraction angiography.

Vital signs initially suggested hemodynamical instability, with a blood pressure of 63/40, but 102/40 mmHg after fluid resuscitation. Laboratory work-up was remarkable for a white blood cell count of 18,100 µL, hemoglobin 10.1 g/dL, red blood cell count of 2.82 million cells/µL, and mean corpuscular volume of 100.4 fL. The patient underwent an abdominal CT with contrast, which revealed perihepatic-predominant hemoperitoneum (Figure 1). No definitive masses, abscesses, obstructions, or perforations were identified. The patient was admitted and interventional radiology was consulted due to worsening hemodynamic status. The patient underwent invasive angiography and right hepatic artery embolization to control the bleeding (Figure 2). Super-selective angiography beyond the right hepatic artery was not attempted because of the patient’s destabilization. A few days following the incident, the patient was discharged and advised to restrict strenuous physical activity for three months with scheduled follow-up imaging. A follow-up MRI soon after demonstrated a large subhepatic hematoma with areas of heterogeneous arterial enhancement, which was thought to be related to ischemia from the broad area of embolization, but further imaging was recommended. 

**Figure 3:**
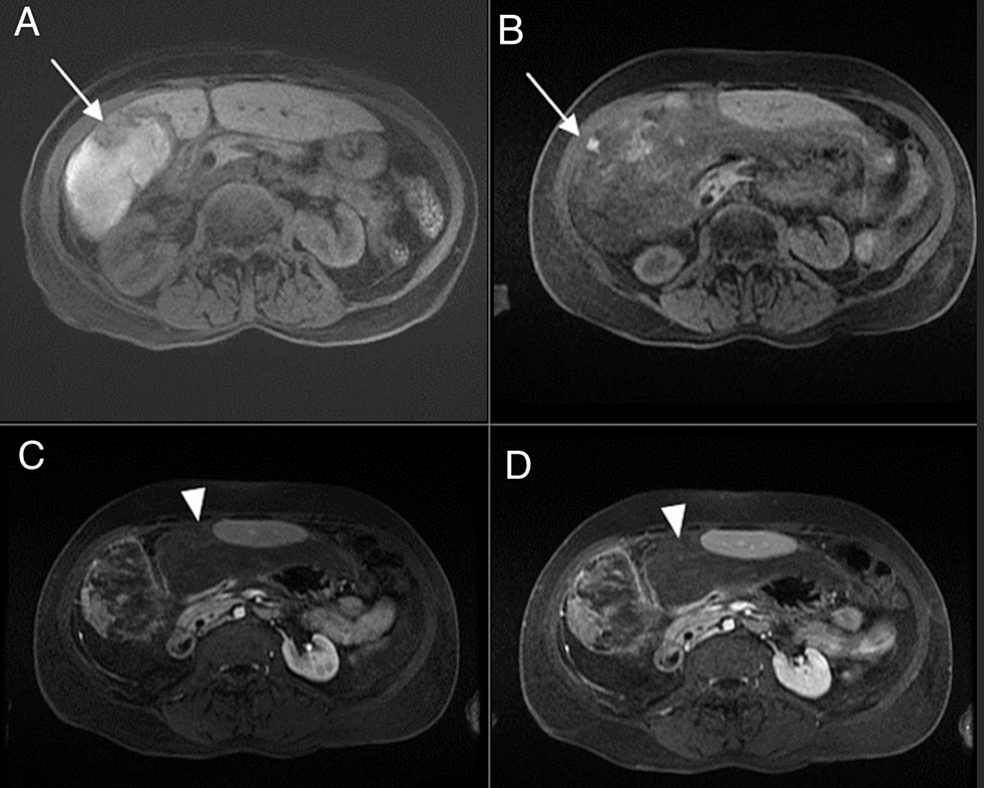
Representative images from follow-up MRI. A) Axial pre-contrast T1-weighted MRI showing nodularity attributed to possible ischemic changes from recent right hepatic artery embolization (white arrow) with subjacent intrinsic bright signal in the subhepatic mass consistent with hematoma. Post-contrast T1-weighted MRI images with arterial (B), portal venous (C), and delayed (D) phases showing abnormal round focus of enhancement and questionable enhancement within the adjacent hematoma suggestive of possible malignancy versus post-embolization changes near the right lobe (white arrow) surrounded by extensive non-enhancing material underneath the left lobe (white arrowhead), also consistent with hematoma. MRI: magnetic resonance imaging.

Repeat MRI (Figure 3) demonstrated acute-on-chronic hemoperitoneum secondary to a peripheral right hepatic lobe mass that demonstrated diffusion restriction in an irregular area of hyperenhancement and washout (an LI-RADS 5 lesion). However, the entire subhepatic mass or hematoma did not demonstrate these features, with fine details including subtraction images being obscured from respiratory motion (not shown).

**Figure 4:**
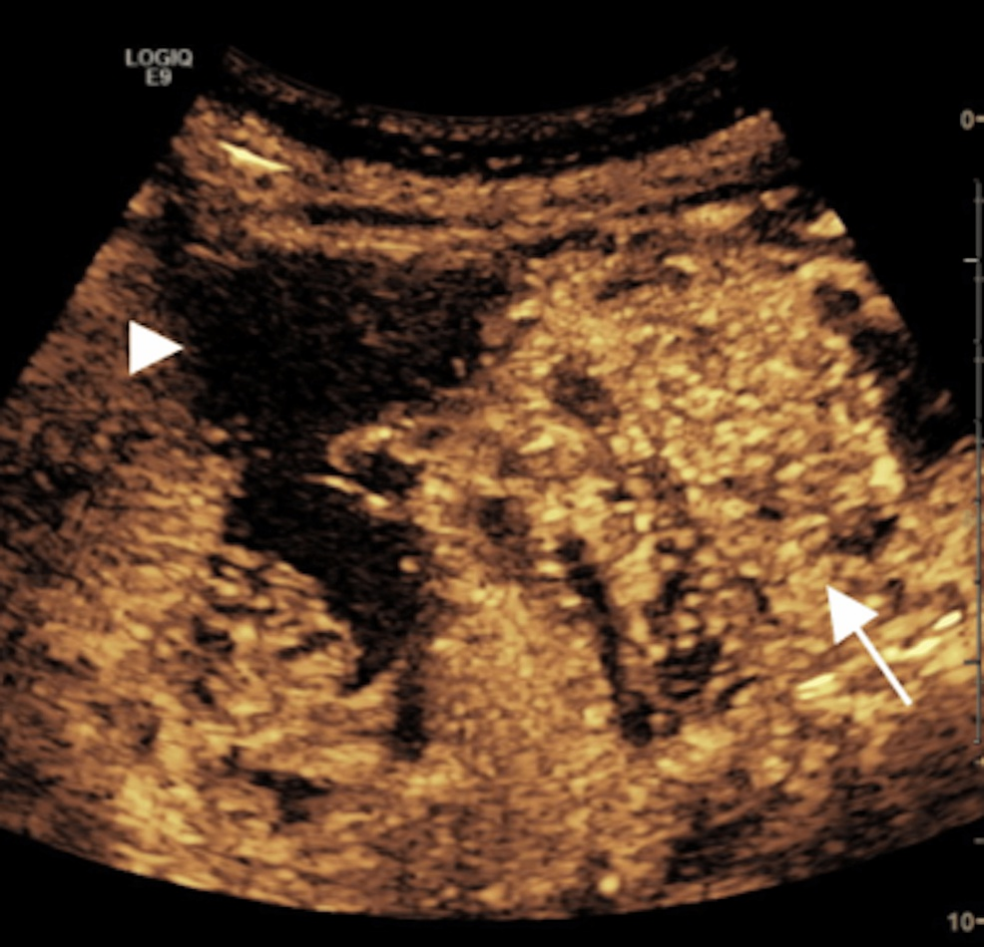
Follow-up CEUS of the abdomen with ultrasound-guided biopsy showing definitive enhancement of the subhepatic mass (white arrow) more than the normal liver (white triangle) suggestive of malignancy. CEUS: contrast-enhanced ultrasound.

CEUS was performed at the time of image-guided biopsy to delineate the target given the irregular appearance of the subhepatic component of the lesion (Figure 4). The subhepatic portion did demonstrate real-time contrast uptake. A biopsy was performed, confirming HCC with the comment of extensive hemorrhagic and necrotic components.

The patient underwent chemotherapy and Yttrium-90 (90Y) radioembolization planning, but ultimately elected hospice. 

## Discussion

HCC is the most common form of primary liver cancer and is now the third leading cause of cancer death [3]. Worldwide, the hepatitis B virus is the leading cause; however, hepatitis C, alcoholic cirrhosis, and non-alcoholic steatohepatitis are important causes as well [4]. CT and MRI imaging are crucial for the diagnosis of HCC and are often able to definitively diagnose HCC without the need for a biopsy [3]. Based on the characteristics of the lesion identified on CT or MRI they are classified from 1-5 in the LI-RADS. The average probability that a lesion represented HCC at the time of imaging ranges from 0% in LI-RADS 1 lesions to 96% in LI-RADS 5 lesions [3]. For LI-RADS 3 and 4 lesions, a biopsy is sometimes recommended to confirm the diagnosis of HCC. A classification of LI-RADS 5 is diagnostic of HCC and biopsy is usually not necessary for confirmation [3]. A more recently implemented imaging modality for the diagnosis of HCC is CEUS. It is not currently as widely used for the diagnosis of HCC, but a meta-analysis published in 2017 showed that it has some advantages over CT and MRI [5].

Our patient had hepatitis B and presented with a ruptured HCC, though this was only clear in retrospect. The initial MRI was only a few weeks after the embolization of the right hepatic artery. This was problematic in the interpretation of lesions supplied by the right hepatic artery, as LI-RADS classification relies on the typical pattern of arterial enhancement. This case raises a question: How soon after arterial embolization (bland, radioembolic, or with chemotherapy) will LI-RADS be useful or applicable? The optimal timing still remains unclear as enhancement from the procedure itself can make it difficult for image interpretation of liver lesions. After repeating CT or MRI to establish a posttreatment baseline, LI-RADS currently recommends follow-up CT or MRI in three-month intervals. However, further research needs to be done to address this over the growing number of cases treated with intra-arterial therapy as discussed next.

The mainstay of treatment for patients with HCC is resection. Resection is ideally performed in patients with HCC without cirrhosis and who at the time of the procedure have adequate liver function and are expected to have good liver reserve after resection. In patients meeting these criteria, survival rates are 70% at five years after resection [3]. Resections may be performed laparoscopically or open and the choice depends on the patient's characteristics. Resections are performed utilizing the liver’s inherent segmental structure with its corresponding eight functional segments [6]. There are two main categories of liver resections, anatomical resections and non-anatomical resections. Anatomical resection involves resecting the tumor, the portal veins draining the tumor, and the corresponding hepatic territory [7]. Non-anatomical resection is defined as the resection of the tumor and a surrounding margin without necessarily adhering to segmental anatomy [6]. For larger tumors, anatomical resection may be associated with better outcomes as compared to non-anatomical resection; however, the currently existing data are inconclusive [7]. For patients with poor liver function and moderate- to low-stage cancer, a liver transplant may be an option [8]. Patients with intermediate-stage unresectable HCC can be considered for liver-directed therapy such as transarterial radioembolization (TARE) with Y90 [9]. Ablation techniques, immunotherapy, and chemotherapies are other possible treatment options [3].

While the patient, in this case, was not a candidate for resection due to the subhepatic/peritoneal disease and never elected to complete her TARE therapy, unusual cases such as this highlight the importance of considering guideline applicability and deviations from normal clinical courses that have robust data supporting guidelines.

## Conclusions

Subcapsular hepatic hematoma can stem from a variety of etiologies, including life-threatening conditions such as rupture of HCC. Depending on the onset and severity, the patient's clinical presentation can take on various forms. Therefore, medical imaging is critical for diagnostic confirmation to guide management. LI-RADS helps categorize focal liver lesions in cirrhotic and chronic hepatitis patients. Following lesion classification, patients may or may not need repeat imaging or image-guided biopsy for further workup. In some instances, such as this patient, subhepatic masses show irregular margin hyperenhancement and washout on CT/MRI. Although LI-RADS 5 lesions do not warrant biopsy, sometimes biopsy is still performed to prevent false-positive diagnoses. Imaging modalities such as DSA can be valuable to localize and detect acute gastrointestinal bleeding; however, hepatic artery embolization prior to CT/MRI/CEUS LI-RADS can impose a barrier to proper HCC characterization. Arterial phase hyperenhancement and washout appearance in the portal venous/delayed phase are the most important distinguishing features that characterize malignant lesions from benign lesions.

## References

[1] Ward WG Sr, Rougraff B, Quinn R, Damron T, O'Connor MI, Turcotte RE, Cline M (2007). Tumors masquerading as hematomas. Clin Orthop Relat Res.

[2] Liu CJ, Chien RN, Yen CL, Chang JJ (2007). Spontaneous rupture of the liver in a patient with chronic hepatitis B and D. World J Gastroenterol.

[3] Marrero JA, Kulik LM, Sirlin CB (2018). Diagnosis, staging, and management of hepatocellular carcinoma: 2018 practice guidance by the American Association for the Study of Liver Diseases. Hepatology.

[4] Llovet JM, Kelley RK, Villanueva A (2021). Hepatocellular carcinoma. Nat Rev Dis Primers.

[5] Zhang J, Yu Y, Li Y, Wei L (2017). Diagnostic value of contrast-enhanced ultrasound in hepatocellular carcinoma: a meta-analysis with evidence from 1998 to 2016. Oncotarget.

[6] Aragon RJ, Solomon NL (2012). Techniques of hepatic resection. J Gastrointest Oncol.

[7] Kang KJ, Ahn KS (2017). Anatomical resection of hepatocellular carcinoma: A critical review of the procedure and its benefits on survival. World J Gastroenterol.

[8] Morise Z, Kawabe N, Tomishige H (2014). Recent advances in liver resection for hepatocellular carcinoma. Front Surg.

[9] Kallini JR, Gabr A, Salem R, Lewandowski RJ (2016). Transarterial radioembolization with Yttrium-90 for the treatment of hepatocellular carcinoma. Adv Ther.

